# Present progress in biomarker discovery of endometrial cancer by multi-omics approaches

**DOI:** 10.1186/s12014-025-09528-6

**Published:** 2025-04-26

**Authors:** Yuhao An, Quanxin Feng, Li Jia, Xinrui Sha, Tuanjie Zhang, Linlin Lu, Rui Wang, Bin Bai

**Affiliations:** 1 Department of Gastrointestinal Surgery, Xijing Hospital of Digestive Diseases, Xijing Hospital, Fourth Military Medical University, Xi’an, Shanxi 710032 China; 2 Pingshan Translational Medicine Center, Shenzhen Bay Laboratory, Shenzhen, Guangdong 518020 China; 3 Faculty of Pharmacy and Pharmaceutical Sciences, Monash University, Parkville, VIC 3052 Australia; 4 Joint Laboratory for Translational Cancer Research of Chinese Medicine of the Ministry of Education of the People’s Republic of China, International Institute for Translational Chinese Medicine, Guangzhou University of Chinese Medicine, Guangzhou, Guangdong 510006 China

**Keywords:** Endometrial cancer, Biomarker discovery, Multi-omics

## Abstract

Endometrial cancer (EC), a prevalent and intricate disease, is associated with a poor prognosis among gynecological malignancies. Its incidence rising globally underscores the urgent need for biomarkers detection in both research and clinical settings. Over the past decade, we’ve witnessed rapid advancements in biological methodologies and techniques. A multitude of omics technologies, encompassing genomic/transcriptomic sequencing and proteomic/metabolomic mass spectrometry, have been extensively employed to analyze both tissue and liquid samples derived from EC patients. The integration of multi-omics data has not only broadened our understanding of the disease but also unearthed valuable biomarkers specific to EC. This review encapsulates the recent progress and future prospects in the application of multi-omics technologies in EC research, emphasizing the potential of multi-omics in uncovering novel biomarkers and enhancing clinical assessments.

## Introduction

Endometrial cancer (EC) is a type of cancer that originates from the inner epithelial layer of the uterus, which is rising in both incidence and associated mortality [1, 2]. Worldwide, approximately 420,368 women are diagnosed with endometrial cancer each year, and it is estimated that this cancer results in the death of about 97,723 women (https://www.wcrf.org/cancer-trends/endometrial-cancer-statistics/). When considering all stages collectively, the overall survival rate for 5 years is approximately 80% [3]. Depending on the clinical symptoms of the endocrine-metabolic condition and hyperestrogenic manifestations, Bokhman systematically classified endometrial cancer into two categories for the first time in 1983 [4, 5]. Type I, also known as endometrioid endometrial carcinoma (EEC), is the most common type, accounting for about 80% of all cases. It has low grades and a favorable prognosis, associated with excess estrogen, obesity, and endometrial hyperplasia. On the opposite, “Type II”, also named uterine serous carcinoma, is a higher-grade non-endometrioid tumor, accounting for about 10%, associated with atrophic endometrium, exhibiting more aggressive behavior and has a worse prognosis [6]. Endometrial clear cell carcinoma (ECCC) is a relatively rare type of endometrial cancer, typically less than 6% of all endometrial cancers [7]. It shares the features of the former two types, such as it is associated with atrophic endometrium, showing aggressive development and worse prognosis like type II, and the characteristics at the immunohistochemical and molecular levels like type I [8]. The rest type of EC is carcinosarcoma, which is a relatively rare and highly invasive type of endometrial cancer, only account for around 3% of endometrial cancers [9]. The definition as a framework instructs the study of teaching and scientific of endometrial cancer continuously. With the advancement of molecular techniques over the decades, multiple-dimensional characteristics have been used to supplement EC’s classification definition. The Cancer Genome Atlas Research Network released a study that profiling EC at the genomic, transcriptomic, and proteomic levels. Based on the mutations and histological features, they classified EC into four types, such as POLE (a catalytic unit of DNA polymerase epsilon) ultramutated, accounting for 7.3% (17 of 232); MSI (microsatellite instability) hypermutated, accounting for 28.0% (65 of 232); low-copy-number, accounting for 38.8% (90 of 232); and high-copy-number, accounting for 25.9% (60 of 232) [10]. Early diagnosis is an ideal strategy to improve the low survival rate, evaluating the risk of EC becoming malignant or recurring, measurable indicators of abnormal biological states or dysfunctional conditions such as biomarkers or molecular features could be extremely useful [10–13]. The alteration detection at the early stage holds the potential to refine the comprehensive classification system for improved accuracy in diagnosing malignancy, as well as to enhance the effectiveness of corresponding treatments. Herein, we offer an overview of the role multi-omics technologies play in biomarker discovery and discuss the prospective strategies for the early diagnosis of EC.

### Multi-bio-layer omic data for prognosis of EC

**Fig. 1:**
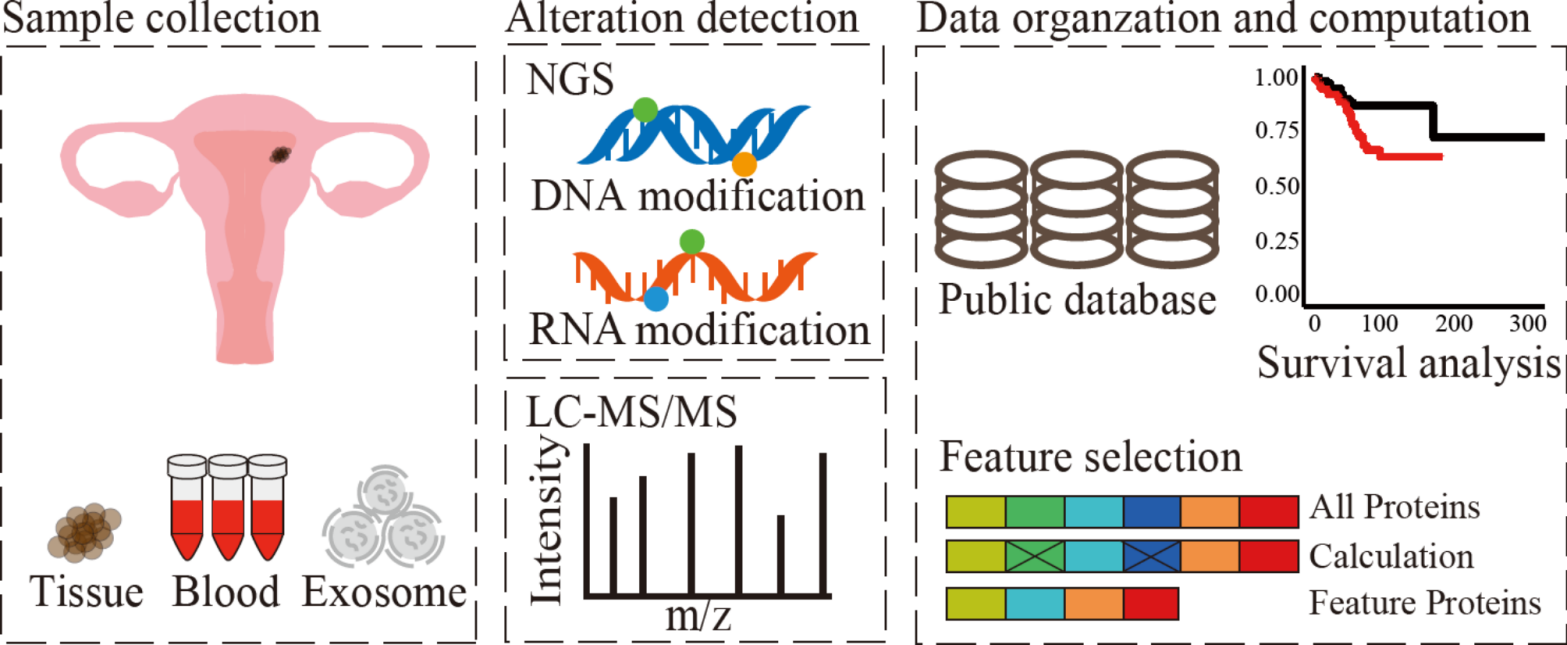
Omic data generation and computation from various sources at different expression levels

### Sample source

As Fig. 1 shows, advances in novel technique development have enabled the use of many types of samples in biomarker discovery. Tissue is the traditional and primary sample for detecting certain gene products specifically expressed in the tumor, facilitating the identification of specific biomarkers [14]. The testing based on tissue biopsy provides a definitive diagnosis of cancer by observing the morphology of the cells and identifying the malignant features. Although tissue biopsy is a standard for diagnosing and studying cancer, it does have several limitations. Because tumors are heterogeneous, tissue biopsy might not describe the entire cancer profiling. The poor repeatability of sample collection complicates this condition further. Additionally, puncturing to collect biopsies may facilitate cancer cell metastasis and elevate the risk to patients’ lives [15]. In EC studies, the sampling procedure also poses challenges with approximately 6–33% of cases being unsuitable for pathological diagnosis due to the limited volume of target areas [16]. Liquid biopsy refers to the testing of blood or bodily secretions for the presence of cancer cells [17], which is a minimally invasive approach without surgical procedures. The low cost and convenience make biofluids ideal samples for tumor research. Compared with tissue biopsies, biofluids focus on the localized area of the tumor, liquid samples could provide information on the whole body condition by continuous monitoring without any invasive operation [18], helping doctors assess the effectiveness of the therapy, and make the necessary adjustments. The screening approaches for tumor-derived factors present in a variety of body fluids [19], such as blood [20], cervicovaginal fluid [20, 21], urine [22, 23], uterine lavage fluid [24], abdominopelvic washing, and ascites [25], have been used in the study and diagnosis of EC, measuring the characteristics of different levels. In the clinic, blood stands as the most commonly tested specimen for liquid biopsy. Except for that, several other bodily fluids have been shown high valuable as a non-invasive sample source. Cervicovaginal fluid is a complex blend of secretions from the uterus, cervix, and vagina, which has been used for biomarker discovery of gynecological diseases [21, 23]. There are studies indicating that the molecular signatures of endometrial cancer (EC) can be identified in cervical scrapes [26], endometrial brushes [27, 28], vaginal swabs [29, 30], and tampons [31, 32], suggesting that endometrial cell fragments could also be present in urine [22, 23]. Uterine lavage and abdominopelvic washing are procedures in a gynecological study introducing a saline solution into the uterine, abdominal, and pelvic cavities, and collecting the subsequent for detection and evaluation of cancer development. Ascites, a common symptom in late-stage cancer, is a valuable liquid biopsy medium, which often occurs in advanced cancer cases, containing cellular and molecular shed by the tumor. The biofluid contains shed cells [33], cancer cells [25, 34], tissue fragments, and other substances from the endometrial lining [24], such as proteins [35], and exosomes [36], providing a profiling of the tumor microenvironment, offering insights into the progression of abdominal malignancies such as endometrial [25], ovarian [37], pancreatic [38], and gastrointestinal [36] cancers. Exosomes are nanovesicles ranging from 30 to 150 nanometers in size, released by various cell types of both tissue and biofluids, rich in nucleic acids, lipids, and proteins [39–41]. They are extracellular structures enclosed by a lipid bilayer, formed by the outward budding of the plasma membrane and released into the extracellular environment through fusion with the plasma membrane [42], delivering information from tumor cells and various cells to the microenvironment [43–46]. Exosomal compositions reflect the molecular characteristics of the cells of origin and vary depending on distinct dysfunctional status. Consequently, tumor cells release unique exosomal contents, providing specific molecular mediators in extracellular communication [47, 48].

### Data expression levels

Whether tissue, biological fluids, or exosomes [49] from them are important carriers of genetic material, full of various cancer biomarkers. In the advance of high-throughput techniques development, large amounts of omic data were generated and analyzed. The advent of next-generation sequencing (NGS) has significantly enhanced the precision and efficiency of genomic and transcriptomic studies, resulting in tons of discoveries related to genetic factors in cancer. The first major type of ‘omics’ data to be found and measured on the biology and development of cancer came from genomic sequences. Based on NGS, tumor genomic profiling becomes a common tool for classifying different types of cancer and identifying biomarkers that can evaluate and predict disease behavior. Somatic variants during cancer incidents can lead to the development and progression of tumors by affecting genes that control cell growth, division, and death. The distribution of mutation frequencies and types from cancers could be adopted for patient diagnostics and biomarker discovery [50]. Somatic copy number alterations (SCNAs), a type of somatic variant, occur in cancer cells to participate in cancer development by amplifications and deletions of the DNA segments. Through SCNAs, endometrial cancer was classified into different groups with significant prognostics [10], showing the potential of SCNAs as a tool for biomarker detection. Circulating tumor DNA (ctDNA) [51] is a kind of small nucleic acid released into the blood from the apoptosis or necrosis of tumor cells, whose expression level is affected by various immune responses, tumor status, and progression [52].

In contrast to the genome, which offers a relatively stable representation of the genetic characteristics, the transcriptome changes across various tissues, stages of development, and disease conditions [53]. Thus, understanding the variations in the transcriptome is key to knowing how genes respond and adjust under genetic or environmental factor influence. Messenger RNA (mRNA) serves as a template for protein synthesis. The mRNA abundance is often measured to determine the expression of genes associated with cancer. For instance, it has been adopted for prognostic markers of EC, promoting the development of effective targets in EC treatment [54]. In the human genome, the vast majority of genes are transcribed into non-coding RNAs (ncRNAs), which do not translate into proteins but have important biological functions, regulating the initiation and progression of various cancers [55]. Despite ncRNAs not being translated into proteins, there is persuasive evidence that they may act as promising biomarkers for cancer prognosis by regulating other genes [56]. In recent decades, studies of the function, regulatory mechanism, and therapeutic potential of the ncRNAs in EC are rapidly evolving, such as microRNA (miRNA) [57, 58], long ncRNA (lncRNA) [59], and circular RNA (circRNA) [60]. miRNAs are endogenous, small ncRNAs (19–25 nucleotides) that regulate gene expression post-transcriptionally [61], which attaches to matching sequences within specific mRNAs, usually resulting in gene silencing through translational repression or target degradation [62]. miRNAs also connect with EC development, which can regulate the expression of genes involved in adhesion, migration, and invasion [63]. Based on this fact, several studies presented that miRNA could be the biomarker to predict the prognosis of EC [64, 65]. Zhou et al. conducted miRNA biomarker identification using the TCGA database and developed a linear regression model to assess the progression of EC. In their study, 26 miRNAs were found to have characteristics of two groups with different immune response conditions [66]. lncRNAs are longer than 200 nucleotides, also widely involved in a range of physiological activities [67]. Jiang et al. identified 1,931 expressed lncRNAs and established a subgroup classification depending on the discovery, providing the feature patterns to measure the risk of malignancy of EC [68]. Compared with lncRNAs, circRNAs are also more than 200 nt, but with a ring structure. The circular structure makes them more stable than linear RNAs, acting as competitive endogenous RNAs or as “microRNA sponges” to bind microRNAs, and preventing them from binding their target mRNAs and regulating gene expression in this way. Due to their ring-like structure stability and cell-type-specific expression patterns, circRNAs have been investigated as potential biomarkers for EC [69, 70].

Proteins, as the executors of gene functions, play a vital role in cellular activities and biological processes. The proteome refers to the complete set of proteins present in a cell, tissue, or organism at a specific time and under particular conditions. Proteomics is the large-scale study of proteomes, focusing on the comprehensive analysis of all proteins produced by an organism, system, or biological context. Mass spectrometry (MS) emerges as a precise technique for comprehensive proteomic studies, facilitating the measurement of features and alterations in proteomes. Proteomics complements genomics and transcriptomics by providing information about the actual functional molecules within the cell and can uncover changes in protein expression, modifications, localization, and interactions that occur in cancer states. There are two primary methods for acquiring proteomic data in the data generation process: data-dependent acquisition (DDA) and data-independent acquisition (DIA). The DDA method traditionally selects peptides based on the highest intensity signals for mapping to a pre-defined database [71]. While this approach enhances the credibility of the results, its stochastic selection process can lead to the loss of valuable information regarding low-abundant peptides [72]. In contrast, the DIA strategy is built upon the development of high-speed scanning techniques, which collect fragments of individual peptides across a series of mass windows, providing comprehensive peptide profiling with high reproducibility [71]. Both methods were employed for biomarker discovery in endometrial cancer (EC). For instance, in Wang’s study [73], 20 patients with endometrial lesions and 7 healthy women were enrolled for specimen acquisition, leading to the identification of 7 biomarker candidates for EC using LC-MS/MS with a DDA strategy. Meanwhile, Jamaluddin’s team collected 63 tumor biopsies from 20 patients to profile the expression features of EC using the DIA strategy [74].

The term “epigenetics” was originally coined by Conrad Waddington in 1942 to describe heritable changes in phenotype that occur without alterations in the DNA sequence. Benefiting from the development of various global omic techniques, the complexity and plasticity of epigenetics have become increasingly visible to us. Epigenetics encompasses a variety of reversible modifications to DNA, proteins, and RNA, usually happening on chromatin, that work together to regulate gene expression and cellular function [75]. As the primary mechanism of carcinogenesis at the epigenetic level, hypermethylation at site-specific DNA could silence tumor suppressor genes, while global hypomethylation can lead to chromosomal instability and oncogene activation [76]. In a study, researchers collected urine samples from 42 endometrial cancer (EC) patients and 46 healthy controls, identifying three DNA methylation markers that demonstrated high predictive performance for EC [77]. It has been shown that DNA methylation in urine is an effective source for EC biomarker discovery. Chromatin serves as an instructive DNA scaffold that responds to external factors for DNA regulation. As the principal component of chromatin, histones play crucial functions through post-translational modifications (PTMs). Acetylation, phosphorylation, glycosylation and other modifications as PTMs have gotten more attention for their diverse roles in the regulation of gene expression, protein structure, and molecular interactions. Some studies show that changes in the PTMs of proteins that are essential for prognosis and cell proliferation can cause tumorigenesis [39, 78]. Since protein is the basic functional element of most biological processes, its abundance and PTMs have been studied to provide deeper insights into disease, which is valuable in identifying diagnostic and prognostic markers for EC [79, 80]. In comparison with the modifications of DNA and proteins, relatively few reports indicate that mRNA undergoes extensive chemical modifications that could alter its function [81]. Post-transcriptional modification refers to the various biochemical modifications that RNA undergoes after transcription and before translation. These modifications are essential for the stability, localization, and function of RNA molecules. N6-methyladenosine (m6A) modification is widely identified in mRNA, which regulates gene expression across several processes related to tumor proliferation, invasion, and epithelial-mesenchymal transition [82]. By studying these modifications, scientists can gain a deeper understanding of the mechanisms of gene regulation and their roles in biology and disease [83].

### Publicly available sources

Many types of omic data have been gathered and used for the establishment of publicly available databases, accelerating the step of biomarker discovery processes. As well-known, The Cancer Genome Atlas (TCGA) is a vast collection of genetic data, combining over 11,158 samples across 32 cancer types [84]. This resource compiles detailed genetic information based on microarrays and next-generation sequencing methods, including RNA sequencing (RNAseq), microRNA sequencing (miRNAseq), DNA sequencing (DNAseq), SNP-based platforms, array-based DNA methylation sequencing, and reverse-phase protein array (RPPA) [85]. According to the TCGA database, Miao’s team comprehensively investigated the variations of EC specimens at the genomic, transcriptomic, and epigenetic levels, resulting in 80 cancer-testis antigens (CTAs) genes more abundant in EC than in normal tissues. Among them, highly expressed TTK protein kinase (TTK), critical in driving EMT and chemoresistance, was significantly linked with lower survival in EC patients, showing the potential of being biomarker [86]. The Gene Expression Omnibus (GEO, http://www.ncbi.nlm.nih.gov/geo/) is a global public database committed to the storage of high-throughput microarray and next-generation sequencing functional genomic datasets. In Wu’s study, TCGA and GEO were integrated to build and validate a prognostic model for EC malignancy prediction [87]. Proteomics offers a way to link gene alterations and cellular physiology. The Clinical Proteomic Tumor Analysis Consortium (CPTAC) provides the first multi-omics database that integrates mass spectrometry (MS)-based global proteomics data, expanding the support for TCGA samples [84, 88]. Combining the clinicopathological data from the TCGA, GEO, and CPTAC, Zhang found the gene products of S100A2 highly expressed in EC tissue at both the mRNA and protein levels, related to IL-17 signaling pathways [89]. The Human Protein Atlas (HPA) focuses on protein expression, distribution and localization, which aims to map all the human proteins in cells, tissues, and organs using the integration of various omics technologies, providing antibody-based imaging, mass spectrometry-based proteomics, clinical and histopathological details [90]. Through HPA database, Zhu measured the functions of TIMM8A in EC, resulting it could be a biomarker to predict the efficacy of anti-PD-L1 therapy [91].

### Bioinformatics analysis of biomarker discovery

**Fig. 2:**
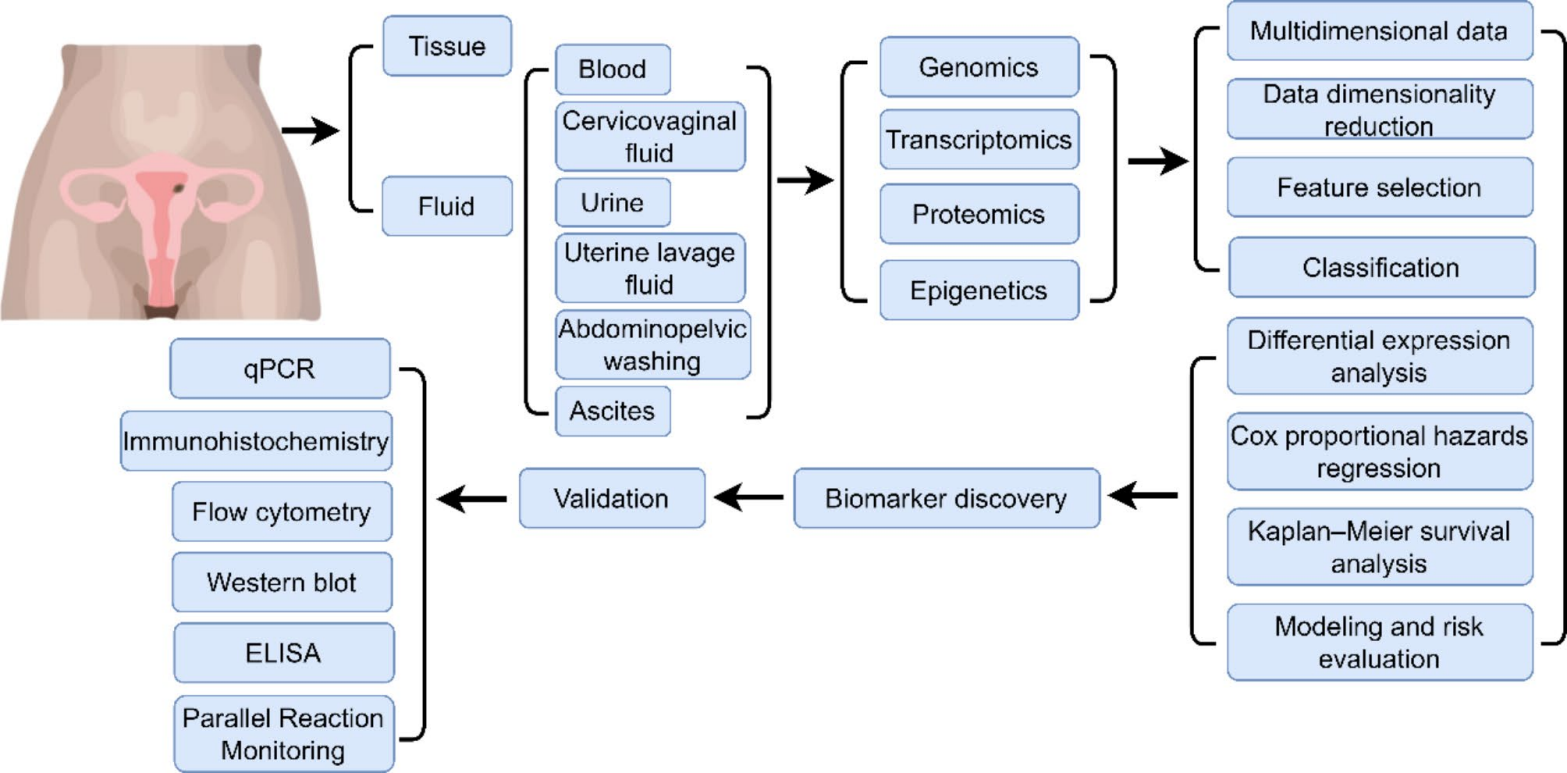
Schematic representation of multi-omics approaches toward diagnosis in endometrial cancer

The emergence of numerous databases has made integration analysis possible, which is valuable in disease research, providing a more comprehensive view of the biological systems by considering the complex interactions between different types of molecular data. According to the definition of “proteomic biomarker” from the Mischak study [92], we could describe “biological marker” at different expression levels as specific gene products that are linked with specific conditions. Biomarkers could be used to assess the risk of disease malignancy, declining the death ratio of the patients. Understanding the clinical questions is the most important issue that needs to be considered in biomarker discovery. Under the clinical question’s definition, the experimental workflow will be built properly, including sample type selection, case-control design, matched collection procedure, and decision of research direction underlying biological processes associated with clinical symptoms. Sufficient clinical data and matched information are beneficial to biomarker discovery. Detailed clinical information accompanying the samples is essential, such as sex, age, disease subtypes, disease stage, diagnosis, long-term follow-up data, etc. The factors could affect biomarker levels and have value in the predictive process, reflecting the correlations with disease progression or treatment response.

Here, a general workflow of biomarker discovery has been shown in Fig. 2. With advances in high-throughput techniques, the abundance of omics data generated makes it possible to combine machine learning algorithms, enhancing this field development. The application of machine learning in biomarker discovery is greatly impactful. Machine learning can process and analyze large-scale biomedical datasets, identifying complex patterns and associations in data and mining potential biomarker candidates [93, 94]. The performance of algorithm models is often improved with the increase in training data size. More data can help the model capture more complex patterns, get a better generalization ability, and improve the robustness to face the noise, performing more reliable predictions. Bioinformatics data have unique characteristics, such as high dimensionality, complexity, heterogeneity, noise, and multiscale nature, which make building the data analysis workflow a huge challenge.

To counterwork the difficulty of high dimensional data display, data dimensionality reduction was used to reduce the number of variables in a dataset through a mathematical transformation while preserving data features as much as possible. Principal Component Analysis (PCA) [95], Linear discriminant analysis (LDA) [96], t-Distributed Stochastic Neighbor Embedding (t-SNE) [97], and Uniform Manifold Approximation and Projection (UMAP) [98] are commonly used in data dimension reduction (Table 1). They help people better understand data patterns and distribution by visualization, promoting the development of analytical strategies.

**Table 1:** Comparison of four dimensionality reduction algorithms

Algorithms	Advantages	Disadvantages
PCA[95]	Simple and easy to understand, high computational efficiency, retains global structure	Linear assumption, sensitive to outliers, limited interpretability
LDA[96]	Good classification performance, suitable for labeled data	Linear assumption, sensitive to high-dimensional data
t-SNE[97]	Excellent local structure preservation, suitable for nonlinear data	High computational complexity, sensitive to parameters, difficult to interpret
UMAP[98]	Preserves both global and local structure, high computational efficiency, flexibility	Sensitive to parameter selection, limited interpretability

Feature selection is a fundamental phase in numerous machine learning workflows, aiming to streamline the task by discarding extraneous factors that might impact model performance. The gene products were treated as features to distinguish different types of samples in studies [99]. High-quality feature data directly influence the accuracy and trustworthiness of biomarker identification. It improves data utilization efficiency and bolsters the validity and clinical applicability of research findings. There are many algorithms (Table 2) available for feature selection, such as Random Forest (RF) [100] algorithm, Boruta [101] (based on RF algorithm), Recursive Feature Elimination (RFE) [102], and Least Absolute Shrinkage and Selection Operator (LASSO) [103].

**Table 2:** Comparison of four feature selection algorithms

Algorithms	Advantages	Disadvantages
RF[100]	Handles large datasets with high dimensionality well, robust to overfitting, can handle both classification and regression tasks	Can be computationally intensive and slow to train, less interpretable, may require tuning of hyperparameters for optimal performance
Boruta[101]	Comprehensive feature selection by comparing actual features with shadow features, robust to noise and missing values, automated process with minimal human intervention, suitable for various data types	High computational cost due to multiple model training, sensitive to parameters of the underlying random forest, may select redundant features in highly correlated datasets, less effective with small sample sizes
RFE[102]	Effective for selecting important features, can improve model performance by reducing overfitting, works well with various types of models	Computationally expensive, especially with large datasets, may not perform well with highly correlated features
LASSO[103]	Performs both variable selection and regularization, can handle multicollinearity by shrinking coefficients, produces sparse models, making interpretation easier	Sensitive to the choice of regularization parameter, may exclude important variables if they are correlated with others, assumes a linear relationship between predictors and the response

Clustering or classification algorithms (Table 3), such as Hierarchical clustering (HC) [104], K-means [105], k-Nearest Neighbor (KNN) [106, 107], and Support Vector Machine (SVM) [107, 108], play an important role in feature selection, as they can group genes with similar expression patterns, which helps reduce the dimensionality of the data, extract representative features, and simplify subsequent analysis. Grouped gene products with similar functions could reveal functional modules and potentially related biological pathways. Meanwhile, classifying samples into different subtypes based on their molecular characteristics is helpful in specific pattern identification under different biological conditions, providing new perspectives to study the disease mechanism and promotes the discovery process of biomarkers.

**Table 3:** Comparison of four algorithms

Algorithms	Advantages	Disadvantages
HC[104]	Does not require the number of clusters to be specified in advance, produce the dendrogram, can capture complex cluster shapes	Computationally expensive, sensitive to noise and outliers, difficult to determine the optimal number of clusters from the dendrogram
K-means[105]	Simple and easy to implement, efficient for large datasets with a known number of clusters	Requires the number of clusters to be specified in advance, sensitive to initial centroid placement and outliers
KNN[106, 107]	Simple and intuitive, effective for small datasets and when the decision boundary is complex, no explicit training phase	Primarily used for classification, computationally expensive during prediction, sensitive to irrelevant features and the choice of distance metric
SVM[107, 108]	Effective in high-dimensional spaces, can model complex decision boundaries using kernel functions, robust to overfitting in high-dimensional space	Requires careful tuning of parameters (e.g., kernel choice, regularization), computationally intensive, especially with large datasets

Differential expression analysis is essential for discerning notable differences in gene expression, protein levels, or other molecular measurements across varying conditions, such as between states of health and illness, and distinct treatments with temporal intervals. DESeq2 [109] is one of the algorithms for differential expression analysis in RNA-seq data, which estimates the variance and mean of gene expression through a model to identify differentially expressed genes, using shrinkage estimation for dispersions and fold changes to improve the stability and interpretability of estimates. edgeR [110] is a Bioconductor software package for examining the differential expression of replicated count data, which was constructed by an overdispersed Poisson [111] model and Empirical Bayes [112, 113] methods, improving the reliability of inference. limma [114] is another Bioconductor software package, suitable for microarray and RNA seq data, using empirical Bayesian methods and linear models to evaluate gene expression differences, emphasizing statistical power and multivariate analysis.

Survival information is invaluable in evaluating disease prognosis and treatment response. It can be adopted to measure the relationship between molecular characteristics and disease progression. To address the need, the Cox proportional-hazards model (COX) [115] and Kaplan-Meier survival analysis (KM) [116] have been widely utilized. Cox model is a statistical technique that allows researchers to determine whether there exists a statistically significant association between the feature patterns and the clinical events occurring over time. In contrast to the Cox model, the KM method emphasizes the assessment of survival time, facilitating the graphical representation of survival outcomes for patients with specific feature patterns, complementing the insights from the Cox model. In Li’s research [99], univariate Cox regression analysis was employed to assess the impact of individual genes on the incidence of endometrial cancer. Meanwhile, the findings from the KM method were combined to provide Death-associated protein kinase-3 (DAPK3) and recombination signal binding protein for immunoglobulin kappa J region (RBPJ) as the biomarker candidates through Boruta and differential expression analysis. According to biomarker candidates of EC and multi-variate Cox regression, they built a linear model for the patient’s prognosis. The methods mentioned above are used both integratively and partially in the discovery of tumor biomarkers.

### Biomarker candidate validation and application

Experimental validation is crucial because it could confirm the true connections between biomarkers and clinical outcomes, guarantee the biomarkers’ reliability, reproducibility, specificity, sensitivity, and biological relevance, and ensure the biomarker is not only statistically associated with the disease but is also practically useful for diagnosis, prognosis, or monitoring in a real-world clinical setting. The selection of experimental methodologies should depend on the types of gene products, ensuring appropriate validation consistent with the specific molecules under study.

Quantitative Polymerase Chain Reaction (qPCR), also known as real-time PCR, which is used to amplify and quantify a targeted DNA molecule. qPCR has several advantages, such as high sensitivity and specificity, real-time monitoring, high-throughput, and versatility [117]. qPCR can detect small amounts of target sequences and differentiate variants of similar nucleic acid sequences accurately. In Chen’s study [118], qPCR has been utilized to identify TMEFF2 as the biomarker of EC. Immunohistochemistry (IHC) is a technique for detecting specific proteins through antigen antibody reactions in tissue sections. It typically uses fluorescent or enzyme labeled antibodies, combined with colorimetric reactions or fluorescent signals, to directly visualize the expression and localization of target proteins on tissue slices. Through IHC and qPCR, CCL25, CXCL10, CXCL12, and CXCL16 were validated for EC biomarker discovery [119]. Flow cytometry is employed to examine the physical and chemical characteristics of cells in a fluid suspension, including cell size, complexity, and the presence of specific surface markers or intracellular molecules. This technique enables the evaluation of thousands of cells per second, providing detailed information about individual cells. Using this method, ERRα was measured and validated in EC tumorigenesis in Su’s research [120]. Western Blot (WB) is commonly used to detect specific proteins from tissue homogenate or extract. The high sensitivity of this technique facilitates the identification of specific target proteins for research objectives. Blendi.et al. [121] and Małgorzata.et al. [122] used western blot as a validation method to confirm the ABRACL and HLA-G respectively as potential biomarkers in EC. Enzyme-Linked Immunosorbent Assay (ELISA) is a widely used biochemical technique for detecting and quantitating specific proteins or molecules in a sample. It has high specificity and sensitivity due to antigen-antibody reaction, providing exceptional efficiency, and allowing for simultaneous analyses without the need for intricate sample pre-treatment procedures. Nonetheless, it’s important to note that ELISA assays typically focus on measuring one protein target per assay, constraining the simultaneous analysis of multiple biomarkers. In the context of endometrial cancer research, ELISA measures diverse biomarkers associated with the disease, such as tumor-associated antigens, cytokines, growth factors, or hormones [123]. For example, leptin was measured by ELISA and IHC in the study about EC progression [124]. Parallel Reaction Monitoring (PRM) is an advanced mass spectrometry-based technique used for targeted proteomics analysis This method enables the simultaneous detection and quantification of multiple proteins or peptides of interest in complex biological samples. PRM offers higher sensitivity, selectivity, and multiplexing capabilities compared to traditional shotgun proteomics approaches [125], making it an invaluable technique in the field of translational research and personalized medicine. After comparing the EC and non-EC tissues, Martinez-Garcia identified PERM, CADH1, SPIT1 and OSTP as the potential biomarkers by PRM [126]. Biomarkers are commonly used to define and identify different molecular subtypes, which are associated with specific biomarkers that can predict patient prognosis and response to treatment. Several biomarkers have been used for prognostic, predictive of treatment response (Table 4) in EC studies through those methods [127, 128].

**Table 4:** Biomarkers of endometrial cancer

Biomarkers	Methods
CCL25, CXCL10, CXCL12, and CXCL16[119]	qPCR, IHC
ERRα[120]	Flow cytometry
ABRACL[121] and HLA-G[122]	Western Blot
leptin[124]	ELISA, IHC
PERM, CADH1, SPIT1 and OSTP[126]	PRM
TMEFF2[118, 129]	qPCR

## Conclusion and discussion

Despite the significant advances made by multi-omics technologies in uncovering potential biomarkers in recent years, the transition from discovery research to clinical practice continues to present widespread challenges. To design experiments scientifically, a clear grasp of medical issues and clinical symptoms is crucial, as the foundation for all steps of biomarker research. Multi-omics techniques generate and analyze large amounts of data. Selecting proper sample types and matched data could help find the relationships between the molecular features and the corresponding disease symptoms, optimizing the data integrative analysis. The advances in high-throughput technologies and the implementation of novel approaches combining machine learning algorithms make multi-layer comprehensive analysis widely performed in biomarker discovery. Multi-omics aims to detect the complex pathophysiology of disease incidents, identifying risk factors and diagnostic cancer biomarkers. The complex cell signaling pathways leading to cancer development rely on the cumulative impact of changes across various expression levels. Therefore, a comprehensive understanding of the various cancer types and stages necessitates the adoption of a multi-omics approach, accelerating the progression of translation into clinical practice and establishment into precision medicine.

## Data Availability

No datasets were generated or analysed during the current study.
